# *In vivo* selection for spine-derived highly metastatic lung cancer cells is associated with increased migration, inflammation and decreased adhesion

**DOI:** 10.18632/oncotarget.4416

**Published:** 2015-06-10

**Authors:** Xiaopan Cai, Jian Luo, Xinghai Yang, Huayun Deng, Jishen Zhang, Shichang Li, Haifeng Wei, Cheng Yang, Leqin Xu, Rongrong Jin, Zhenxi Li, Wang Zhou, JianDong Ding, Jianjun Chu, Lianshun Jia, Qi Jia, Chengjun Tan, Mingyao Liu, Jianru Xiao

**Affiliations:** ^1^ East China Normal University and Shanghai Changzheng Hospital Joint Research Center for Orthopedic Oncology, Department of Orthopedic Oncology, Changzheng Hospital, The Second Military Medical University, Shanghai, P. R. China; ^2^ Shanghai Key Laboratory of Regulatory Biology, Institute of Biomedical Sciences and School of Life Sciences, East China Normal University, Shanghai, P. R. China; ^3^ The Key Laboratory of Adolescent Health Assessment and Exercise Intervention of Ministry of Education, East China Normal University, Shanghai, P. R. China; ^4^ Department of Orthopedics, Binhu Hospital, The First People's Hospital of Hefei City, Hefei, P. R. China; ^5^ Department of General Surgery, The Fifth People's Hospital of Qinghai Province, Qinghai, P. R. China; ^6^ Center for Cancer and Stem Cell Biology, Alkek Institute of Biosciences and Technology, Texas A&M University Health Science Center, Houston, TX, USA

**Keywords:** lung cancer, bone metastasis, spine metastasis, A549

## Abstract

We developed a murine spine metastasis model by screening five metastatic non-small cell lung cancer cell lines (PC-9, A549, NCI-H1299, NCI-H460, H2030). A549 cells displayed the highest tendency towards spine metastases. After three rounds of selection *in vivo*, we isolated a clone named A549L6, which induced spine metastasis in 80% of injected mice. The parameters of the A549L6 cell spinal metastatic mouse models were consistent with clinical spine metastasis features. All the spinal metastatic mice developed symptoms of nerve compression after 40 days. A549L6 cells had increased migration, invasiveness and decreased adhesion compared to the original A549L0 cells. In contrast, there was no significant differences in cell proliferation, apoptosis and sensitivity to chemotherapeutic agents such as cisplatin. Comparative transcriptomic analysis and Real-time PCR analysis showed that expression of signaling molecules regulating several tumor properties including migration (MYL9), metastasis (CEACAM6, VEGFC, CX3CL1, CST1, CCL5, S100A9, IGF1, NOTCH3), adhesion (FN1, CEACAM1) and inflammation (TRAF2, NFκB2 and RelB) were altered in A549L6 cells. We suggest that migration, adhesion and inflammation related genes contribute to spine metastatic capacity.

## INTRODUCTION

Lung cancer is currently the most commonly diagnosed cancer and the leading cause of cancer deaths, causing more than one million deaths each year in the world [1]. Like many other cancers, lung cancer-related deaths are mainly linked to metastases rather than to the primary tumor in most patients [2-4].

Despite increasing knowledge in cancer biology, the treatment efficacy of distant stage lung cancer has not improved significantly over the past decade and the overall five-year survival rate is just 3.6% [5]. Bone is the most common site of lung cancer metastasis. Up to 40% of lung cancer patients with advanced stage disease have bone metastases [5]. The most common bone metastasis site of lung cancer is the spine which accounts for more than 50% of bone metastasis cases [6]. Spinal metastases are the major source of pain and disability in patients with cancer [7, 8]. Among the malignancies that metastasize to the spine, lung cancer is one of the most aggressive, with a 1-year survival rate of ∼ 22% [7]. Patients with spinal metastases experience severe pain, pathologic bone fractures, spinal cord compression, and hypercalcemia, which entail poor quality of life and decreased survival rates as well as substantial health care costs [9, 10].

However, the molecular mechanisms underlying the bone tropism of lung cancer metastases remain poorly understood, and our ignorance is even worse for spine metastasis. This gap in knowledge is due to difficulty in generating ideal animal models that more closely reflect tumor biology in humans. Current bone metastatic mouse models of lung cancer are mainly evaluated by metastatic lesions in limb bones rather than by spinal metastases [8, 11], thus they do not fully recapitulate the clinical characterizes of spine metastasis in lung cancer. Therefore, there is considerable scientific interest in finding more appropriate animal models for spine metastasis.

In this report, we successfully developed a novel spine metastasis mouse model after *in vivo* screening. This model has an 80% efficiency of spine metastasis as determined by bioluminescence imaging (BLI). Moreover, the spine metastasis lesions were confirmed by X-ray, Micro-CT, MRI and histopathological staining. The cells, which can induce spine metastasis, had increased migration, invasion, and decreased adhesion. At the molecular level, RNA-seq analysis demonstrated that many critical regulators of cancer functions were changed. These genes included tumor markers, as well as genes that control tumor migration, metastasis, cell adhesion and inflammation. Thus, our findings support the notion that this novel molecular signature contributes to aggressive spine colonization by a mechanism involving the cooperative action of cell migration, adhesion and inflammation.

## RESULTS

### *In vivo* selection of spine metastatic cell line

**Figure 1:**
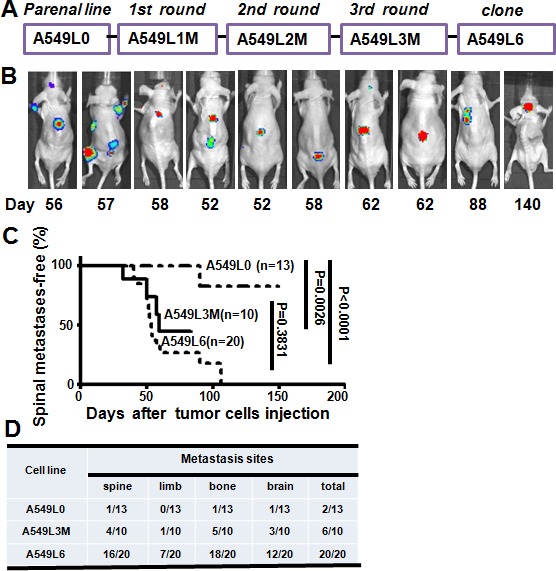
Establishment of the spinal metastasis model **A.** *In vivo* selection scheme for the spinal metastatic subpopulations from the A549 lung adenocarcinoma cell line. Luciferase-labeled A549 cells (A549L0) were intracardially inoculated into nude mice. Spinal metastases were monitored by BLI after injection. Tumor cells were extracted from spinal metastases and selected by G418, then reinoculated after expansion in culture. After three rounds of *in vivo* selection, ten subclones including A549L6 were selected and re-inoculated into mice to evaluate their metastatic phenotype. **B.** Nude mice were intracardially inoculated with 1×10^5^ A549L6 cells and monitored using bioluminescence imaging (*n* = 20). 10 representative mice are shown. **C.** Kaplan-Meier analysis of spinal metastases-free survival according to BLI. **D.** The target organs of the original A549 cells (A549L0), the 3^rd^ round spine metastasis cancer cells (A549L3M), and the spinal metastatic cancer cells (A549L6) in nude mice.

*In vivo* selection has proven an effective approach to isolate organ-specific metastatic subpopulations from heterogeneous cancer cell lines [12-14]. To develop a highly spine metastatic non-small cell lung cancer cell line, we choose five widely-used metastatic non-small cell lung cancer cell lines (PC-9, A549, NCI-H1299, NCI-H460, H2030) and transfected each with a luciferase reporter gene for metastasis analysis *in vivo*. The spine metastatic activity was assessed by *in vivo* bioluminescence imaging. Our data showed that all of the five cell lines induced mild metastasis and the A549 cell line (A549L0) displayed the most prominent spine metastasis lesions by 90 days (Supplemental Figure 1A and data not shown). Therefore, we choose the A549 cell line for further *in vivo* selection. We extracted the cancer cells from the spine as the 1^st^ round cells (A549L1M) and expanded them in culture for another two rounds of *in vivo* section (Figure 1A). To our surprise, three rounds of *in vivo* selection generated a subline (A549L3M) with only a mildly increased (up to 40%) spine metastatic rate (Figure 1D). Because the A549 cell line was a heterogeneous cell population [15, 16], we chose 10 A549L3M clones with the highest luciferase expression for the spine metastasis evaluation. One clone (A549L6) generated spinal metastasis in 80% of injected mice (Figure 1B and 1D, *n* = 20). The spine metastases were initially detected by bioluminescence imaging at 40 days after inoculation of 1×10^5^ A549L6 cells (Figure 1C). The spine metastases rate is significantly higher than that of both the original cells (A549L0) and the 3^rd^ round cancer cells (A549L3M) (Figure 1C and 1D).

### Confirmation of the spine metastases

**Figure 2:**
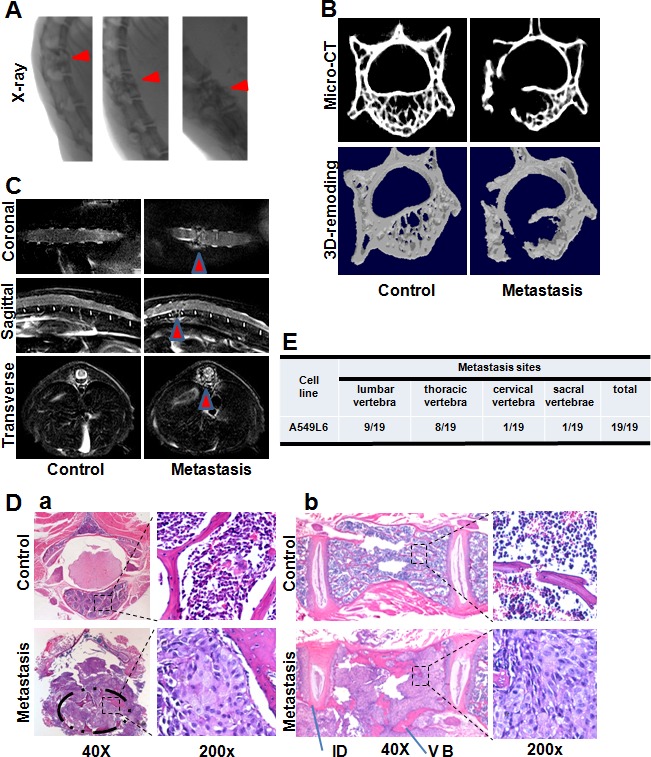
Assessment of the spinal metastasis lesions **A.** Representative spine X-ray radiography imaging of mice 60 days after inoculation with the A549L6 cells. Metastatic lesions are indicated by red arrows. **B.** Representative Mirco-CT imaging (top) and 3D-remodeling imaging (bottom) of mice 50 days after inoculation with A549L6 cells. **C.** Representative T2-Weight MRI imaging at 50 days after inoculation with A549L6 cells. **D.** Representative HE staining imaging of cross sections **a.** and longitudinal sections **b.** of thoracic vertebrae metastasis lesions. **E.** The metastatic vertebra regions observed in mice injected with the spine specific metastasis cancer cells (A549L6).

To further characterize the spine metastasis, we used 4 separate approaches (X-ray, Micro-CT, MRI and histopathological section) to analyze the spine metastases in the A549L6 inoculation mice (Figure 2A-2D). All of the experimental paradigms showed that the cancer cells induced dramatic spine metastases, which occured in different vertebrae including lumbar vertebra (9/19), thoracic vertebra (8/19), cervical vertebra (1/19) and sacral vertebrae (1/19) (Figure 2E). Interestingly, the cancer cells did not destroy the intervertebral discs even though the whole vertebral body was infiltrated by cancer cells (Figure 2D). This phenomenon is consistent with the clinical features of spinal tumors in human patients [17, 18].

### Clinical parameters of the spinal metastatic models

**Figure 3:**
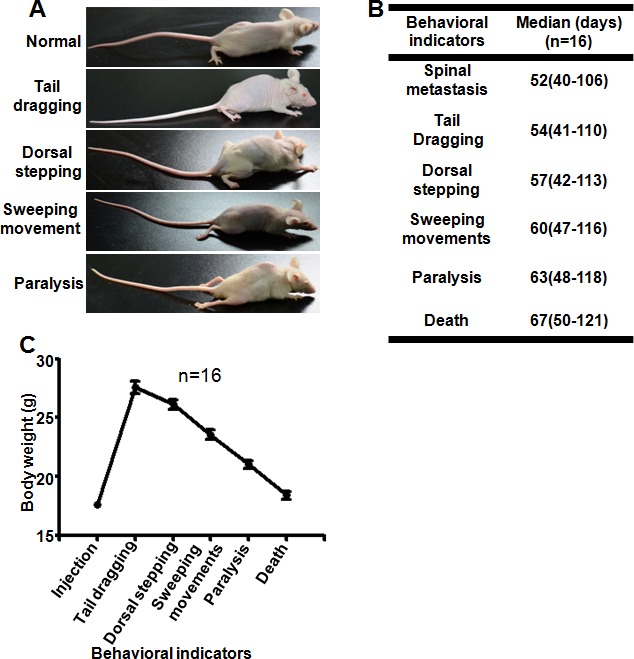
Neurological, functional and survival assessment of spinal cord compression A Representative photographs of 4 key indicators of nerve compression symptoms after cancer cell metastasis to the spine: tail dragging, dorsal stepping, sweeping movement, and paralysis. **B**. The median time to each key indicator after A549L6 cell injection. Data in this figure represent mean ± SD (*n* = 16). **C.** The body weight changes of mice inoculated with A549L6 cells at each of the 4 key milestones from tail dragging to paralysis. Data in this figure represent mean ± SD (*n* = 16).

The ideal animal model should mimic the human disease. Spine metastasis in humans often leads to nerve compression symptoms including radicular pain, weakness, paralysis, and finally, death. In mice, there are 4 behavioral indicators of nerve compression progression: tail dragging, dorsal stepping, sweeping movement, and paralysis [7]. In our models, all of the spinal metastatic mice developed symptoms of nerve compression after 40 days (Figure 3A and 3B). The median time to first incidence of tail dragging, dorsal stepping, hindlimb sweeping, and paralysis was 54, 57, 60, 63 days after injection of A549L6 cells, respectively (Figure 3B). These results are similar to the clinical nerve compression symptoms of spinal metastatic cancer that include pain, numbness and irreversible loss of neurologic function [19, 20]. The mouse body weight also dramatically decreased from 27.59 ± 2.06 g to 21.01 ± 1.33 g (*n* = 16) as the disease progressed from tail dragging to paralysis (Figure 3C), which was consistent with the clinical features as well [20].

### Biological differences between the original cancer cells and the highly spinal metastatic cancer cells

**Figure 4:**
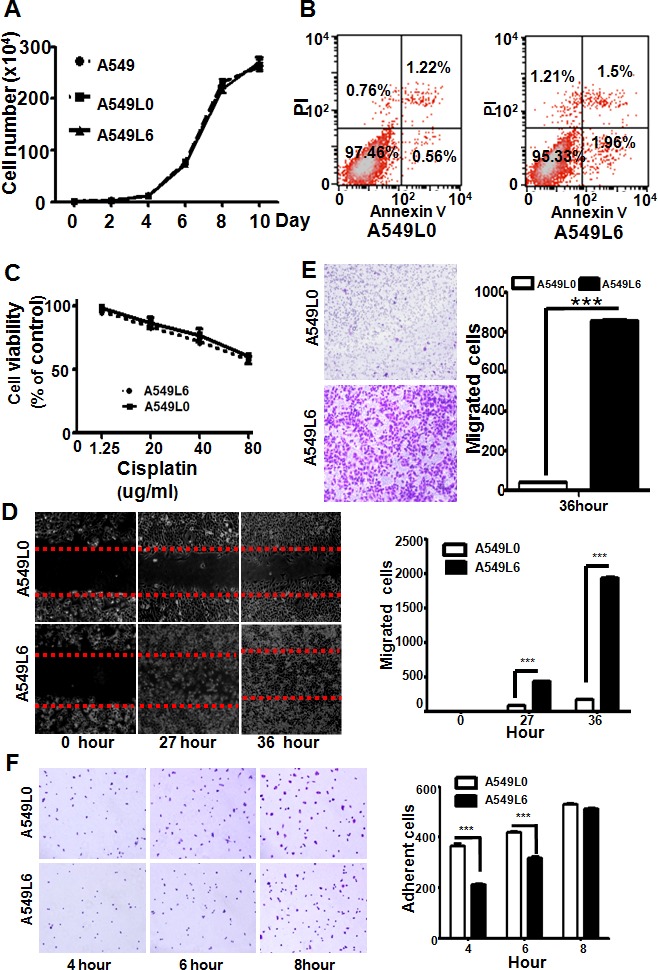
Comparison of biological features between A549L0 and A549L6 cancer cells **A.** No significant differences among the A549, A549L0, and A549L6 cell growth as determined by cell number counting. A549, A549L0, and A549L6 cells were seeded into 6-well cell culture plates at a density of 1×10^4^ cells per well. Cells were counted at the indicated times. Experiments were repeated in triplicate and each experiment was performed three independent times. Results are presented as means ± SEM (error bars). **B.** No significant difference between A549L6 cells and A549L0 cells in cell apoptosis. 95% confluent cells were harvested and stained with FITC Annexin V and Propidium iodide (PI) using the FITC Annexin V Apoptosis Detection Kit, and then detected by FACS. The experiment was performed three independent times. **C.** No significant difference between A549L6 cells and A549L0 cells in cisplatin resistance. A549L0 and A549L6 cells were seeded into 96-well cell culture plates with a density of 4000 cells per well, then treated with indicated cisplatin concentrations for 3 days. Cells were counted at 72 hours post-treatment start. Experiments were performed in triplicate and each experiment was performed independently three times. The means and SEM (error bars) are presented. **D.** Increased migration of the A549L6 cells in wound healing assay. Confluent cells were scratched and the cell migration back into the scratched surface was monitored over 36 hours. Representative images at time points 0, 27 and 36 hours were shown (left). The migrated cells were counted (right). Results are presented as means ± SEM (error bars); ***, *p* < 0.001. **E.** Increased invasion of the A549L6 cells in Boyden chamber transwell assay. 36 hours after seeding indicated cell lines (5×10^4^ cells), invasive cells were stained, photographed (left) and counted (right). Results are expressed as means ±SEM (error bars); ***, *p* < 0.001. **F.** Decreased adhesion of the A549L6 cells. A549L6 cells and A549L0 cells were seeded in gelatin-coated 96-well-plates at a density of 1×10^4^ cells per well and adherent cells were counted at the indicated time points. Representative images (left) and the cells were counted. Results are expressed as means ±SEM (error bars); ***, *p* < 0.001.

Next, we sought to understand how the A549L6 was altered by *in vivo* selection to allow spine metastasis, so we compared the original cancer cell line (A549L0) and the spine metastatic cancer cell line (A549L6) in proliferation, survival, migration, invasion, adhesion, and resistance to cisplatin. Our data showed that there are no significant differences in cell proliferation (Figure 4A), apoptosis (Figure 4B), and sensitivity to chemotherapeutic agents such as cisplatin (Figure 4C) between the two cells. However, the migration (Figure 4D) and invasion (Figure 4E) were dramatically increased and the adhesion of A549L6 cells on gelatin-coated plates was decreased (Figure 4F), suggesting that the increased migration, invasion ability and decreased adhesion contribute towards this highly metastasis model.

### Gene expression signature associated with spine metastatic ability

**Figure 5:**
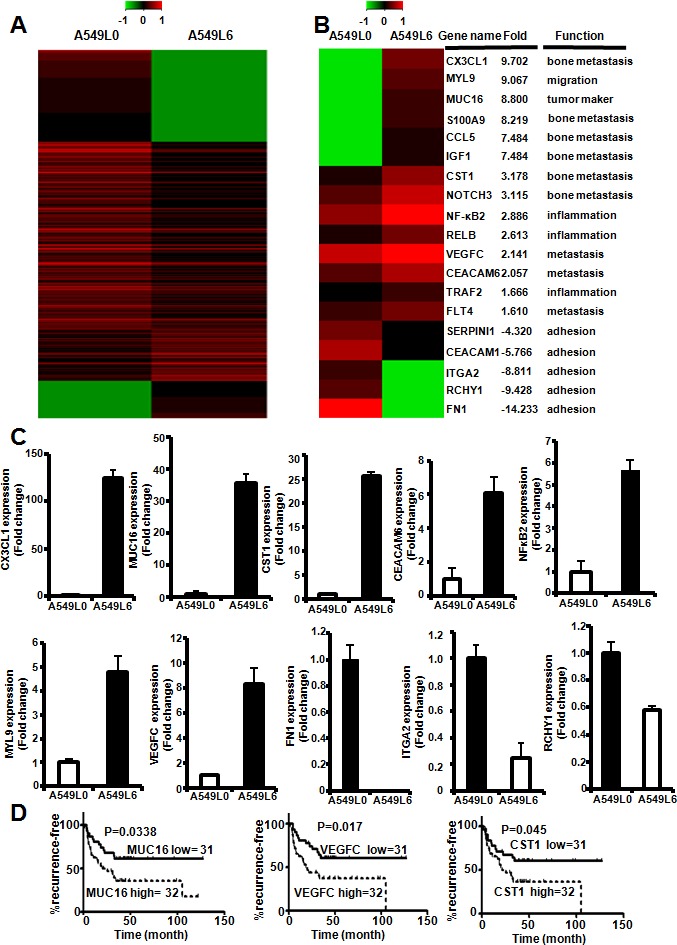
Gene network changes in regulation of tumor migration, metastasis, adhesion and inflammation **A.** Heat map depicting RNA-sequence array expression profile comparing A549L0 cells and A549L6 cells. **B.** Representative genes involved in different cancer functions including migration, metastasis, cancer markers, and NFκB2signaling. **C.** Real-time PCR of 10 selected genes in the A549L0 versus A549L6 cells. The expression levels were in accordance with the RNA-seq results from **A.**. **D.** Kaplan-Meier analysis of recurrence-free survival according to mRNA expression data of selected genes in 63 lung adenocarcinoma patients acquired from microarray analysis (GEO accession # GSE8894). P values were calculated by the two-sided log-rank test.

To identify the mechanism by which A549L6 induces spine metastasis, we performed RNA-seq analyses to find genes that were responsive to spine metastasis. The results showed that 289 genes were up-regulated and 918 genes were down-regulated in the A549L6 cell set (Figure 5A). Functional analysis of these differentially expressed genes revealed that a number of genes involved in many critical aspects of cancer were changed. These genes included tumor markers (MUC16) [21, 22], genes that control tumor migration (MYL9) [23], metastasis (CEACAM6, VEGFC, CX3CL1, CST1, CCL5, S100A9, IGF1, NOTCH3) [24-35], cell adhesion (FN1, CEACAM1) [16, 36, 37], and inflammation (TRAF2, NF-κB2 and RelB) [38, 39] (Figure 5B). Interestingly, some genes such as CX3CL1, CST1, CCL5, S100A9, IGF1, NOTCH3 were related to bone metastasis [26, 28, 29, 31-34]. To confirm the RNA-seq results, we selected 12 genes which represented different functions for validation by real-time PCR. The data were consistent with the RNA-seq results that all of the 12 genes were significantly changed in A549L6 cells (Figure 5C and supplemental Figure 1B). The prognostic value analysis also showed that the MUC16(CA125), CST1 and VEGFC high expression group had significantly shorter recurrence-free survival as compared with the low expression group (Figure 5D). All of the data suggested that migration, metastasis, adhesion and inflammation related genes might contributed to the development of spine metastatic capacity by A549L6 cells.

## DISCUSSION

The skeleton is one of the most common sites of metastasis in lung cancer, in which the vertebral column is the most frequently affected region [6]. To date, the mechanisms of lung cancer metastasis to the spine have been poorly understood. This gap in knowledge is due to the lack of an appropriate animal model. In this study, we developed a novel highly spinal metastatic mouse model which primarily affected the spine and can be sensitively tracked by non-invasive, real-time BLI. More importantly, the spinal metastasis progression clinically correlated with nerve compression symptoms. We also found increased migration, invasion and decreased adhesion in the spine metastasis lung cancer cells. In recent years, several new models of non-small-cell lung cancer (NSCLC) for bone metastasis have been described. These include models for large cell carcinoma (NCI-H460) [8] and lung adenocarcinoma (PC9 and H2030) [11], but these models mainly focused on limb bone metastasis, and they have not been evaluated in their clinical features. One group established a nude mouse model that utilized intravertebral implantation of PC14 lung tumor tissue to establish spinal metastases [7]. This model had the advantages of developing consistent and reproducible numbers of metastases in a relevant location, and neurologic symptoms occurred within a relatively short timeframe. However, it suffered from the fact that this method bypasses the metastatic process of cancer cells transitioning through the circulatory system, which makes this model poorly suited for researching the molecular mechanisms of metastasis. Furthermore, the operation process itself stimulates bone remodeling and results in secondary complications [40], thus the positive effects of spinal metastasis in that model is problematic due to potential surgical confounding.

To date, the mechanisms and pathways that mediate lung cancer metastasis to the spine are poorly understood. Our sequence analysis results suggest that many critical cancer functions were changed in the highly metastatic cell line, including tumor markers (MUC16) [21, 22], genes that control tumor migration (MYL9) [23], metastasis ( CEACAM6, VEGFC, CX3CL1, CST1, CCL5, S100A9, IGF1, NOTCH3) [24-35], cell adhesion (FN1, CEACAM1) [16, 36, 37], and inflammation (NF-κB2, TRAF2, RelB) [38, 39]. These gene sets are consistent with our findings of increased migration, invasion and decreased adhesion in the spine metastasis cell line. Interestingly, the inflammation related genes NF-κB2, TRAF2 and the metastasis related gene CX3CL1 are correlated with the NF-κB signaling pathway. NF-κB signaling is strongly correlated with bone metastaisis [41]. Furthermore, tumor-initiating cells in human prostate cancer exhibit increased NF-κB signaling [42]. Further investigation are required in evaluation whether cancer cells with bone or spine metastatic capability may be tumor-initiating cancer stem cells.

Finally, here we present a process for developing additional organ-specific metastatic sublines from additional primary lung tumor cell lines. In the current bone metastasis models of human lung cancer, various lung cancer cell lines derived from metastatic lesions of patients have been successfully used to establish distant-organ metastasis models. For example, PC9 and H2030 were extracted from lymph nodes of lung adenocarcinoma patients [11], and NCI-H460 was extracted from a pleural effusion [43]. However, the metastatic competence of cell lines derived from primary lung cancer are not yet defined. In our research, we successfully established the spinal metastasis model using the A549 cell line which was established from a localized epithelial lung adenocarcinoma rather than a metastatic lesion [44]. In addition, we also found that almost all A549L6 cells are refractile while both A549L0 and A549 contain only a small fraction of refractile cells (Supplemental Figure 1C). These data suggest that the primary tumor A549 cells also contain highly metastatic cells that can be screened by *in vivo* selection. Therefore, we speculate that some single-cell progenies derived from A549 might also exhibit highly organ-specific metastatic properties.

In conclusion, we present a novel highly spinal metastatic lung cancer mouse model that reflects the course of disease progression in human patients, with potential value in determining the mechanisms of metastatic spread as well as in evaluating novel therapeutics targeting metastatic disease.

## MATERIALS AND METHODS

### Cell line

The A549 (human lung adenocarcinoma), NCI-H460 (human large cell carcinoma) and NCI-H1299 (human lung adenocarcinoma) cell lines were obtained from the American Type Culture Collection. The PC9 (human lung adenocarcinoma) and H2030 (human lung adenocarcinoma) cell lines were a kind gift from Dr. ji (Chinese Academy of Sciences, Shanghai, China P.R).

### Transfection and selection of cell line

A549 cell line was cultured in DMEM medium (Invitrogen, Carlsbad, CA) containing 10% fetal bovine serum (Invitrogen, Carlsbad, CA). NCI-H460, NCI-H1299, PC9 and H2030 cell lines were cultured in RPMI-1640 medium (Invitrogen, Carlsbad, CA) containing 8-10% fetal bovine serum (Invitrogen, Carlsbad, CA).

A549, NCI-H1299, NCI-H460, H2030 and PC-9 cells were transfected with plasmids co-expressing the firefly luciferase gene and G418 resistance gene using lipofectamine 2000 (Invitrogen, Carlsbad, CA). Transfected cells were then selected for G418 resistant stable cell lines. Surviving subpopulations were detected for bioluminescence in PBS supplemented with 150ug/ml D-luciferin (AOK Chem, Shanghai) on a Xenogen IVIS-200 system (Caliper Life Sciences, Hopkinton, MA). All cell lines were cultured under standard culture conditions.

### Wound healing and transwell migration assays

For the wound healing assay, cells were cultured in a 12-well plate. When 80% confluent, cells were starved for 12 h. Then, a wound was scratched in the center of the cell monolayer by a sterile plastic pipette tip. The debris was removed by washing with PBS twice. After 27h and 36h, the wound was photographed under an inverted microscope. For the transwell assay, 5×10^4^ cancer cells suspended in medium without FBS were plated on each upper chamber and the bottom chambers were filled with 600 μL medium containing 10% FBS. After 36 hours, the upper chambers were fixed with 4% paraformaldehyde and stained with 1% crystal violet. Then, non-invasive cells were removed gently using cotton swabs and invasive cells were imaged using an Olympus inverted microscope.

### Growth curve and apoptosis assays

Cell growth was monitored by cell counting on the indicated days after seeding. Briefly, cells were plated in 6-well-plates at a density of 1×10^4^ cells per well and cell numbers were counted on days 2, 4, 6, 8 and 10, respectively. Day 0 was considered the day after plating. For the cellular apoptosis assay, confluent cells at day 8 were harvested and stained with FITC Annexin V and Propidium iodide (PI) using the FITC Annexin V Apoptosis Detection Kit (BD, Franklin Lakes, NJ), and then detected by FACS. The cells double negative for PI and FITC were counted as healthy cells.

### Cisplatin-resistance assay

Cisplatin-resistance was determined by the Sulforhodamine B (SRB) (Sigma, St Louis, MO) assay as described previously [45]. Briefly, A549L0 or A549L6 cells were plated in 96-well-plates at a density of 4×10^3^ cells per well. After 24h, both cells were treated with the indicated concentrations of cisplatin for 72 hours. Cell viability was determined by SRB assay and the optical density (OD) at 515 nm was recorded using a VERSAmax microplate reader (Molecular Devices).

### Adhesion assay

The cell adhesion assay was performed as described previously [46]. Briefly, 96-well plates were precoated with 0.25% gelatin and washed with sterile PBS. 1×10^4^ cells in 100μL DMEM were added to each well and incubated in standard culture conditions. At 4, 6 and 8 hours post-plating, unattached cells were gently removed and attached cells were fixed with 4% paraformaldehyde and stained with 1% crystal violet. Adherent cells were then counted and photographed under a bright field microscope.

### Animal tumor model and *in vivo* spinal metastasis cell line selection

4-6 week old male BALB/c nu/nu mice were purchased from Sino-British Sippr/BK Lab Animal Co, Ltd (Shanghai, China). The number of mice used for A549L0, A549L3M, A549L6 was 13,10,20 repectively. The animal experiment of A549L0 and A549L3M were performed in one time, and that of A549L6 was performed in two independent times. All animal experiments were performed in accordance with animal protocol procedures approved by the Ethics Committee of East China Normal University. A total of 1×10^5^ cancer cells were re-suspended in 0.1 mL PBS and then injected into the left ventricle of mice via a percutaneous approach as previously described [14, 47]. *In vivo* selection of highly metastatic cancers was performed as previously described [14]. Briefly, to isolate tumor cells from the spine metastatic lesions, mice with lesions detected by BLI were sacrificed, and the affected vertebrae were separated from the body. After muscle were removed, tumor cell suspensions were prepared by mechanical dispersion and incubated with collagenase type IV (Sigma, St Louis, MO) for 2 hr under continuous shaking at 37°C. Recovered cells were selected in complete medium supplemented with 0.5-0.8 mg.mL^−1^ G418 (Calbiochem, Darmstadt, Germany). Following expansion *in vitro*, tumor cells were re-injected into the left ventricle of nude mice. When spine metastatic lesions were formed, the mice were killed and tumor cells in the vertebrae were again harvested for culturing.

### Bioluminescence imaging and neurological functional assessment

*In vivo* distant-organ metastasis was monitored by bioluminescent imaging (Caliper Life Sciences, Hopkinton, MA) as previously descripted [48]. Briefly, to detect metastases of A549 cancer cells stably expressing luciferase at indicated days, D-luciferin potassium salt (AOK Chem, Shanghai) was injected intraperitoneally into the anesthetized mice at a dose of 150mg/kg. After 10 min, the mice were placed into the IVIS Imaging System. Imagines were recorded with an exposure time of 5 minutes. Bioluminescence was assayed and photons per second were quantified using software (Living Image 3.2, Caliper). Radiographs were also used to confirm the metastatic lesions in the spine (Carestream, Kodak). Motor features were monitored for neurological functional according to the method described by Tatsui CE et al [7], where motor features are graded to four key milestones: tail dragging, dorsal stepping, sweeping movement and paraplegia. Mouse body weight changes were also recorded daily.

### Histopathology analysis

The spine tissues were harvested and immediately fixed in 10% paraformaldehyde (PFA) for 12 hours, and then embedded in paraffin. All spine tissues were decalcified with 10% EDTA for 2 weeks before they were embedded in paraffin. 4 mm thick sections of the tissues were prepared and stained with haematoxylin and eosin.

### Micro-CT and MRI evaluation

The selected spine samples harvested from euthanized mice were placed in a scanning holder, and then scanned using a micro-computed tomography device (Skyscan 1076, Bruker Micro-CT NV, Antwerp, Belgium). After scanning, the two-dimensional (2D) and three-dimensional (3D) models were reconstructed and evaluated using the CTAn and CTVol programs (Bruker Micro-CT NV, Antwerp, Belgium). For MRI, the anesthetized mice were placed in a 3.0 T MRI scanner (Magnetom Verio, Siemens, Germany ) in the prone position with their tails straight and the T2-weighted images were obtained.

### RNA-sequences array of transcriptome and identification of differentially expressed genes (DEGs)

The A549L0 and A549L6 cells were harvested in the exponential growth phase, and the total RNA from each cell line was isolated using TRIzol (Invitrogen, Carlsbad, CA, USA). For the cDNA library preparation, two cDNA libraries were prepared according to the manufacturer's protocol (Illumina, San Diego, CA, USA). The quantification and qualification of sample libraries were performed using the Agilent 2100 Bioanaylzer (Agilent, Santa Clara, CA, USA) and ABI StepOnePlus Real-Time PCR System (Applied Biosystems, New York, USA/Life Technologies, New York, USA ). Each library was sequenced using the Illumina HiSeq™ 2000 (Illumina Inc, San Diego, CA, USA). These two raw sequencing data sets were deposited in the Sequence Read Archive of NCBI (http://www.ncbi.nlm.nih.gov/sra) under accession number SRX1037575.

The gene expression of A549L0 and A549L6 cells was compared and the differential expression values of genes in each group were calculated. FDR < = 0.055 and |log2Ratio| > = 1.60 were selected as cut-off criteria.

### RT-PCR and real-time RT-PCR

Total cellular RNA was isolated from cells using TRIzol Reagent (Invitrogen, Carlsbad, CA, U.S.) as previously described [49]. Two-step real-time quantitative PCR- analysis was performed using SYBR Green Mix (Takara). PCR primers were (Table 1):

**Table 1:** PCR primers

CX3CL1	5′-CTCCGATATCTCTGTCGTGG 3′ (forward) 5′-CTCCAAGATGATTGCGCGTT-3′ (reverse)
MYL9	5′-CATGTTCCTCACCATGTTTG-3′ (forward) 5′-CATGGATGAAACCTGAGGC-3′ (reverse)
MUC16(CA125)	5′-CCATAAGCCTATCCACTGAG-3′ (forward) 5′-ATCAGTTGTTTCAACCAAGG-3′ (reverse)
CST1	5′-CCTTCACTTCGCCATCAG-3′ (forward) 5′-TCTCCCAGGGAACTTCGT-3′ (reverse)
CEACAM6	5′-GGAAATGCTTCTATCCCTGA-3′ (forward) 5′-GTTAGAAGTGAGGCTGTGAG-3′ (reverse)
NFκB2	5′-GACTGTCACTTGGTGATACA-3′ (forward) 5′-TGTCTTCCTTCACCTCTGT-3′ (reverse)
VEGFC	5′-CCTCAGCAAGACGTTATTTG-3′ (forward) 5′-TGGAATCCATCTGTTGAGTC-3′ (reverse)
FN1	5′-AAACACTAATGTTAATTGCCCA-3′ (forward) 5′-TGTTCCTCTGGATTGGAAAG-3′ (reverse)
ITGA2	5′-GAAAGTGCATACAACACTGG-3′ (forward) 5′-AAGTCACCTGTTGTTCTCTC-3′ (reverse)
RCHY1	5′-AAATTCAACATGCCCAACAG-3′ (forward) 5′-ATCTTCCTTTGGACCAATCC-3′ (reverse)
SERPINI1	5′-CAGTCTGGACTGTAGTTTCC-3′ (forward) 5′-TGTAACAGTTTCAAGCCTCC-3′ (reverse)
RCHY1	5′-AAATTCAACATGCCCAACAG-3′ (forward) 5′-ATCTTCCTTTGGACCAATCC-3′ (reverse)
TRAF2	5′-CCGTTGGGGCTTTGTTC-3′ (forward) 5′-TGCCTTCTTCATATATGCCC-3′ (reverse)

### Survival analysis of clinically annotated lung adenocarcinomas

A clinically annotated microarray database (in which the recurrence sites included bone) from the Samsung Medical Center was obtained from GEO (GSE8894) and was used to determine the clinical significance of our selected genes. This data set contains a mix of 63 lung adenocarcinomas and 75 squamous cell carcinomas, and the 63 lung adenocarcinomas were further selected for Kaplan-Meier analysis of recurrence-free survival.

### Statistical analyses

Numerical data are represented as means ± SD (SEM). Statistical differences between the means for the different groups were evaluated with graphpad prism5 using unpaired Student's t test or one-way analysis of variance (ANOVA). Survival curves were performed with graphpad prism5 using the Kaplan–Meier analysis, and the Log-rank test was used to ascertain the the statistical significance (P value). All statistical tests were calculated two-sided with the level of significance at P≤0.05.

## SUPPLEMENTARY MATERIAL FIGURE


