# Supplementary material for: In vivo selection for spine-derived highly metastatic lung cancer cells is associated with increased migration, inflammation and decreased adhesion
Source: Oncotarget. 2015 Jun 10;6(26):22905–17. doi: 10.18632/oncotarget.4416 (PMC4673208; doi:10.18632/oncotarget.4416)
Supplement: Supplementary file 1 [file oncotarget-06-22905-s001.pdf]

In vivo selection for spine-derived highly metastatic lung cancer cells is associated with increased migration, inflammation and decreased adhesion

Supplementary Material

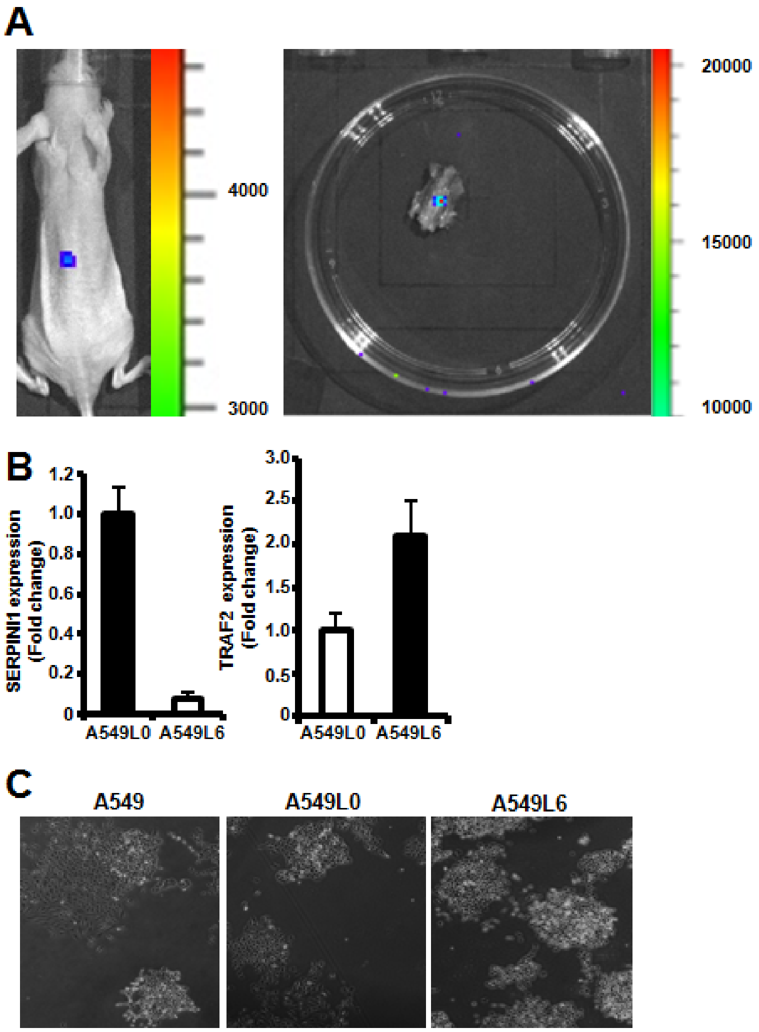

Supplemental Figure 1

(A) The *in vivo* and ex vivo BLI imaging of spine metastasis of A549L0 cells inoculated nude mouse. Nude mice were intracardially inoculated with 1×10^5 A549L0 cells and 1 of 13 mice developed mild lumbar vertebra metastasis monitored using bioluminescence imaging.

(B) qPCR of SERPINI1 and TRAF2 in the A549L0 versus A549L6 cells.

(C) The morphological of A549L6 was different from A549 and A549L0. Almost all A549L6 cells were refractile while A549L0 and A549 just partially contained this kind cell. Representative images of A549 (left), A549L0 (middle) and A549L6 (right).
